# Machine Learning Models for Data-Driven Prediction of Diabetes by Lifestyle Type

**DOI:** 10.3390/ijerph192215027

**Published:** 2022-11-15

**Authors:** Yifan Qin, Jinlong Wu, Wen Xiao, Kun Wang, Anbing Huang, Bowen Liu, Jingxuan Yu, Chuhao Li, Fengyu Yu, Zhanbing Ren

**Affiliations:** 1 College of Physical Education, Shenzhen University, Shenzhen 518000, China; 2 College of Physical Education, Southwest University, Chongqing 400715, China; 3 Physical Education College, Yanching Institute of Technology, Langfang 065201, China

**Keywords:** diabetes, machine learning, lifestyle, data-driven

## Abstract

The prevalence of diabetes has been increasing in recent years, and previous research has found that machine-learning models are good diabetes prediction tools. The purpose of this study was to compare the efficacy of five different machine-learning models for diabetes prediction using lifestyle data from the National Health and Nutrition Examination Survey (NHANES) database. The 1999–2020 NHANES database yielded data on 17,833 individuals data based on demographic characteristics and lifestyle-related variables. To screen training data for machine models, the Akaike Information Criterion (AIC) forward propagation algorithm was utilized. For predicting diabetes, five machine-learning models (CATBoost, XGBoost, Random Forest (RF), Logistic Regression (LR), and Support Vector Machine (SVM)) were developed. Model performance was evaluated using accuracy, sensitivity, specificity, precision, F1 score, and receiver operating characteristic (ROC) curve. Among the five machine-learning models, the dietary intake levels of energy, carbohydrate, and fat, contributed the most to the prediction of diabetes patients. In terms of model performance, CATBoost ranks higher than RF, LG, XGBoost, and SVM. The best-performing machine-learning model among the five is CATBoost, which achieves an accuracy of 82.1% and an AUC of 0.83. Machine-learning models based on NHANES data can assist medical institutions in identifying diabetes patients.

## 1. Introduction

Diabetes prevalence has risen sharply with the aging of the global population and changes in dietary patterns [1]. There will be approximately 537 million diabetics globally by 2022 [1]. Diabetes causes a slew of secondary consequences, including cardiovascular disease [2], kidney damage [3], retinopathy [4], foot damage [5], etc. Diabetes is expected to be the seventh greatest cause of death worldwide by 2030, according to the International Diabetes Federation, while the epidemic is escalating and placing a substantial economic burden on healthcare systems [1].

Previous research has linked diabetes to lifestyle risk factors, such as BMI, unhealthy diet, alcohol, smoking, and physical inactivity [6,7,8,9,10]. For example, Gabriela et al. [7] showed that BMI was consistently related to diabetes incidence in a meta-analysis of 32 studies. Prof et al. [10] reported that smoking, both actively and passively, significantly increased the incidence of type 2 diabetes in a meta-analysis of 88 prospective trials. According to Dolly et al. [6], moderate drinking helps prevent diabetes in both men and women in a meta-analysis of 20 cohort studies. According to Andrea et al. [9], a meta-analysis of 28 prospective studies, higher levels of physical activity were linked to a considerably decreased incidence of diabetes. Andrew et al. [8] conducted a follow-up study of 43,176 Chinese Singaporeans and found that unhealthy dietary habits (dim sum, meat, processed meat, etc.) significantly increased the risk of diabetes.

Machine learning is becoming increasingly popular as a model-building tool in recent years, and it has also been widely discussed in the medical field [11]. Machine learning has demonstrated its powerful predictive capabilities and parallel processing capabilities for handling large numbers of variables. Furthermore, machine learning has derived variable screening mechanisms that can detect and interpret complex relationships between variables. Previous studies have well demonstrated that machine learning may be employed as an effective research method for predicting diabetes. For example, Yu et al. [12] used data from the 1999 National Health and Nutrition Examination Survey database to construct a method for identifying diabetes patients using SVM in 2010. It turns out that support vector machine modeling is a promising classification method that can be used to detect people with common diseases such as diabetes and prediabetes. Sanakal and Jayakumari et al. [13] proposed a system for concatenating and predicting text data using support vector machines and fuzzy C-means (FCM) clustering. The study found that Fuzzy C-means clustering can achieve satisfactory results in predicting diabetes, with an accuracy rate of 94.3%. Dewangan and Agrawal et al. [14] devised a method for diabetes prediction using multilayer perceptrons and Bayesian classification. After using various classification methods and integrating them, the researchers obtained a machine-learning model with a diabetes prediction accuracy of 81.89%. In addition, researchers [15] have conducted a meta-analysis of the ability of machine learning to predict diabetes. It was found that current machine learning algorithms are powerful enough to help clinicians determine whether an individual will develop type 2 diabetes in the future. Other researchers [16] conducted a meta-analysis of machine-learning models for predicting diabetes in pregnant women. It was found that machine learning models were more attractive in predicting diabetes than current traditional screening strategies.

According to existing research, CatBoost, a popular approach introduced in recent years, outperforms other publicly available boosting implementations in terms of quality on various datasets [17]. However, it is unclear whether CATboost can outperform other diabetes predictors. In comparison to other difficult-to-obtain indicators, lifestyle-related indicators are easily accessible and non-invasive. Furthermore, based on current evidence, a substantial association between lifestyle and diabetes has been demonstrated [18,19]. However, only two studies [20,21] included lifestyle variables to train machine learning. The first study [21] omitted the more critical factors of diet and physical activity. The second study [20] predicted cognitive health in adults but did not predict diabetes. In addition, it [21] does not apply the CATBoost and XGBoost machine-learning models that have emerged in recent years, which may explain its machine-learning model’s poor AUC (area under the curveroc). Therefore, our study included significant predictors, a larger number of training people, and CATBoost, XGBoost, and other models that have emerged in recent years to optimize the prediction of diabetes using lifestyle variables. We hope to build a machine learning model that uses a more readily available variable (lifestyle) to predict diabetes more accurately.

This study aimed to train and assess five different machine-learning models utilizing machine-learning-related data from the NHANES (National Health and Nutrition Examination Survey). Based on the papers of other researchers [22,23,24], we hypothesized that CATBoost outperformed the other four machine-learning models in predicting diabetes.

In the method, we introduce the database-related information and the database processing method, the feature variable screening method, five machine learning models, and the contents of the Bagging and Boosting algorithms. In addition, we explain how to evaluate the performance of machine learning models. In the Results section, we show the screening results of feature variables, class imbalance handling of the dataset, and our machine learning model performance. In the Discussion section, we highlight and discuss the differences between our study and previous studies and mention the optimization of our model.

## 2. Materials and Methods

### 2.1. Dataset and Variable Selection

We employed the NHANES dataset that contains the records of 124,821 respondents who lived in the United States during the 1999–2020 pre-pandemic period. A total of 33,332 of the 124,821 respondents had blood glucose data. After excluding data with >10% missing values, 17,833 respondents were included in the present assessment. The NHANES is a research project created to evaluate the nutritional status and the general health of adults and children in the United States. The survey is distinctive in that it combines physical examinations with interviews. The dataset contained 18 diabetes-relevant factors and one outcome that could help establish the connection between lifestyle and diabetes. The outcome variable was blood glucose data (mmol/L) present in NHANES. We judge whether there is diabetes according to the standard proposed by the World Health Organization “fasting blood glucose is seven mmol/L or higher, then it is diagnosed as diabetes [25]”. The influencing factors included essential features like (1) RIAGENDR, (2) RIDAGEYR, (3) RIDRETH, (4) DMDBORN, (5) DMDEDUC, (6) INDFMPIR, (7) DR1TKCAL, (8) DR1TPROT, (9) DR1TCARB, (10) DR1TSUGR, (11) DR1TTFAT, (12) DR1TCHOL, (13) BMI, (14) BPXOSY, (15) BPXODI, (16) LBDGLUSI, (17) ALQ111, (18) SMQ020, and (19) SLD012. A description of the factors influencing the prevalence of diabetes is presented in Table 1.

Feature variables include four categories, namely Demographics Data, Dietary Data, Examination Data, and Questionnaire Data. Demographics Data refers to the basic demographic information of respondents, including RIAGENDR, RIDAGEYR, RIDRETH, DMDBORN, DMDYRUSZ, DMDEDUC, DMDMATZ, RIDEXPRG, and INDFMPIR. The investigators obtained this information through interview interviews. Dietary data is a variety of nutritional information respondents have consumed in the past 24 h, including DR1TKCAL, DR1TPROT, DR1TCARB, DR1TSUGR, DR1TFIBE, DR1TTFAT, DR1TCHOL. The investigators asked the subjects various nutritional intake questions in a private room of the NHANES mobile test center. Examination Data refers to various physical measurements performed on subjects in the laboratory by qualified investigators. These include BMI, BPXOSY, and BPXODI. Questionnaire Data is that respondents use the computer-assisted personal interview system to answer in the NHANES mobile test center, including ALQ111, PA, SMQ020, SLD012, and MHDS.

### 2.2. Class Imbalance Handling

In machine learning data preprocessing, there is often a high imbalance of positive (sample with diabetes) and negative (Samples without diabetes) samples. That is, the number of samples in one category is much larger than in the other [26,27]. Usually, the class imbalance is an obstacle to machine learning, and the proportion of class imbalance also determines the severity of the imbalance to an extent. For example, if a dataset includes 110 samples with positive outcomes and 150 samples with negative outcomes, this can be a minor class imbalance issue. The current machine-learning algorithms can easily resolve this issue. However, if a dataset includes 150 positive-result samples and 1 negative-result sample, it results in a larger class imbalance problem. Class imbalance can be a complex problem for any machine-learning classifier owing to the small number of negative outcome samples participating in the machine-learning model training. If a machine-learning model is trained on data with a large class imbalance, it can become an algorithm with a significant deviation in the prediction results, and the more significant number of classes ends up covering a smaller number of classes as most machine-learning models are devised based on data balance [28,29].

This study adopts SMOTE-NC [30] (Synthetic Minority Over-sampling Technique Nominal Continuous) to deal with unbalanced class data. SMOTE (Synthetic Minority Over-sampling Technique) is a technique that calculates the Euclidean distance of data points in the feature space based on the nearest neighbor. SMOTE alleviates overfitting caused by randomly replicated samples by artificially synthesizing samples. Unlike SMOTE, SMOTE-NC works on datasets containing both numerical and categorical features. Since the dataset in this study mixes categorical data (gender, etc.) and continuous data (BMI, etc.), we employ SMOTE-NC to handle class-imbalanced data.

### 2.3. Feature Variable Filtering

Feature selection plays an important role in classification tasks, affecting the model’s performance [31]. Feature selection aims to identify the most relevant subset of features to improve classification accuracy [32]. The leading cause for using the 18 characteristic variables in Table 1 is to (a) select the variables most commonly used to predict diabetes, (b) add some variables to increase the variety of variables, (c) the impact of observed variables on machine-learning predictions, (d) and stepwise backward selection (using AIC) [33]. AIC is a metric applied to assess how well statistical models fit data. Hiroji Akaike, a Japanese statistician, proposed it in 1974. AIC is based on the idea of entropy and offers a measure for comparing the predicted model’s complexity and the accuracy of data fitting [33]. The stepwise-regression algorithm based on the AIC information criterion can effectively filter the features and avoid the influence of multicollinearity. This study employed the reverse stepwise regression algorithm to screen the characteristic variables. The principle is to first introduce all characteristic variables into an equation and then delete a variable that maximizes the AIC value of the regression equation in each experimental step, followed by the use of the remaining variables toward the reconstruction of the regression equation. Until the AIC value is minimum, the optimal regression equation is obtained [33]. The specific AIC values are depicted in Table 2. In the feature selection process, we introduced the lifestyle variables present in all datasets and used a python program to build code to automate the selection of feature variables. The program selects a new variable and compares the AIC values. If the AIC value decreases, this variable is included (the variable has a model contribution). Otherwise, it is sent out. The variables in Table 2 are the remaining variables, and the corresponding AIC value refers to the change of the AIC value after adding the variable. The Variable in Table 2 refers to the decrease in AIC value after adding this variable, indicating that this variable contributes to the machine learning model, and the AIC value refers to the change in AIC value after adding this variable.

### 2.4. Machine-Learning Classifiers

Classification prediction in machine learning refers to a category of the predicted data (the label of a predicted sample). In our machine-learning research on lifestyle and diabetes, we utilized five different machine-learning classifiers, including XGBoost [34], CATBoost [35], Support Vector Machine (SVM) [36], Random Forest (RF) [37], and Logistic Regression (LR) [38].

XGBoost: Extreme Gradient Boosting is referred to as XGBoost. This machine-learning algorithm is another boosting algorithm presented by Chen and Guestrin [34]. It reduces the overfitting of a model by introducing a regularization parameter as well as improving the speed and efficiency of the model.

CATBoost: In 2017, Yandex, a Russian search engine, developed its CatBoost machine learning framework open source. This algorithm is a member of the Boosting family. The three famous Gradient Boosting Decision Tree (GBDT) artifacts include CatBoost, XGBoost, and LightGBM, all of which are enhanced implementations that fit inside the GBDT algorithm’s framework. Yandex’s CatBoost is a more accurate algorithm than XGBoost and LightGBM, although XGBoost is extensively utilized in the sector and LightGBM significantly increases the computational efficiency of GBDT [35]. CATboost reduces the need for many hyperparameter tuning and reduces the chance of overfitting, which also makes the model more general; in addition, it can match any advanced machine learning algorithm in terms of model performance [35].

SVM: A supervised learning model approach called a support vector machine (SVM) is used to analyze data in classification challenges. The SVM model depicts the training data as various points in space, allowing for the widest possible separation of the data for each specific category in the data mapping process. As a result, it maps the new data onto the same space and predicts the class based on where they fall in an interval [36].

RF: The Random Forest was proposed by Leo Breiman in 2001. It is based on the bagging method and selects subsample-guided data through several iterations of the growing tree. Meanwhile, it also uses a predictor so that each growing book depends on independently sampled random vector values. All trees shared the same distribution [39,40].

LR: Logistic regression belongs to a supervised classification algorithm that predicts the probability of a target label. For LR, variable types are binary, implying that a variable can only have two categories. It is one of the easiest machine-learning models and is frequently used to predict different common binary variables, such as diabetes prediction, heart disease prediction, spam detection, and cancer detection [41,42].

Bagging (bootstrap aggregating): Bagging is a sampling method with a replacement step. It is possible to draw repeated samples. The boosting algorithm extracts the training set from the data set, and each round will extract n training samples from the data set. A total of k rounds of extraction are performed. Finally, k training sets are obtained. The final model uses k training sets to train k models and votes the k models to get the classification results [43].

Boosting: Boosting is an algorithm for assembling weak classifiers into strong ones [44]. Compared with the Bagging algorithm, the data of each training set of boosting is unchanged, and the weights of the samples in the data set (adjusted according to the classification results of the previous round) are changed.

### 2.5. Performance Measurement

To assess the predictive performance of machine learning models, we employed different evaluation metrics models such as sensitivity, accuracy, precision, the AUC, and receiver operating characteristic (ROC). This section lists and explain different formulations used to infer performance metrics for machine learning models.

Accuracy is the most common model evaluation metric in machine learning and is easy to interpret and understand. Accuracy refers to the proportion of correctly predicted samples in the overall sample. Generally, the higher the accuracy, the better the classifier.

The formula used for calculating the accuracy of a machine-learning model is given below:

*Accuracy* = (*TP* + *TN*)/(*TP* + *FP* + *TN* + *FN*) (1)

where *TN*, *FN*, *TP*, and *FP* stand for True Negative, False Negative, True Positive, and False Positive, respectively.

Precision is a measure of the ability of a machine learning model to predict accurately. From the perspective of prediction results, Precision describes how many of the positive results predicted by the two-classifier are true positives, that is, how many of the positives predicted by the two-classifier are accurate.

Sensitivity represents the proportion of all paired positive examples and measures the ability of the classifier to identify positive examples. The higher the sensitivity, the more positive cases are judged as positive, and the patient is judged as a patient without the missed diagnosis. (The numerator represents the correct positive example, and the denominator represents the true positive example of the sample).

Specificity refers to the proportion of identified negative cases out of all negative cases. The higher the specificity, the model will determine as many negative samples as possible to reduce the number of false positives. (The numerator represents the correct negative example, and the denominator is the true negative example of the sample).

The F1 score is the Sensitivity of Precision and Sensitivity. It was introduced to solve the conflict between precision and recall. The higher the precision and recall, the higher the F1 score.

The formula is as follows:

*Precision* = *TP*/(*TP* + *FP*) (2)

*Sensitivity* = *TP*/(*TP* + *FN*) (3)

*Specificity* = *TN*/(*TN* + *FP*) (4)

*F1_Score* = (2 × (*Precision* × *Sensitivity*))/(*Precision* + *Sensitivity*) (5)

The ROC curve, a coordinate-schematic analysis tool, is employed in signal-detection theory to select the best learning model in order to eliminate the second-best model or define the ideal threshold in the same model.

In addition to the abovementioned evaluation metrics, we introduced a confusion matrix to observe the machine-learning model’s predictions more intuitively. Each row of the confusion matrix represents the correct attribution category of the data, and the total number of data in each row represents the number of data instances of that category. Each column of the confusion matrix represents the predicted category, and the total number of each column represents the number of data predicted to be in that particular category.

### 2.6. Overall Process

**Figure 1:**
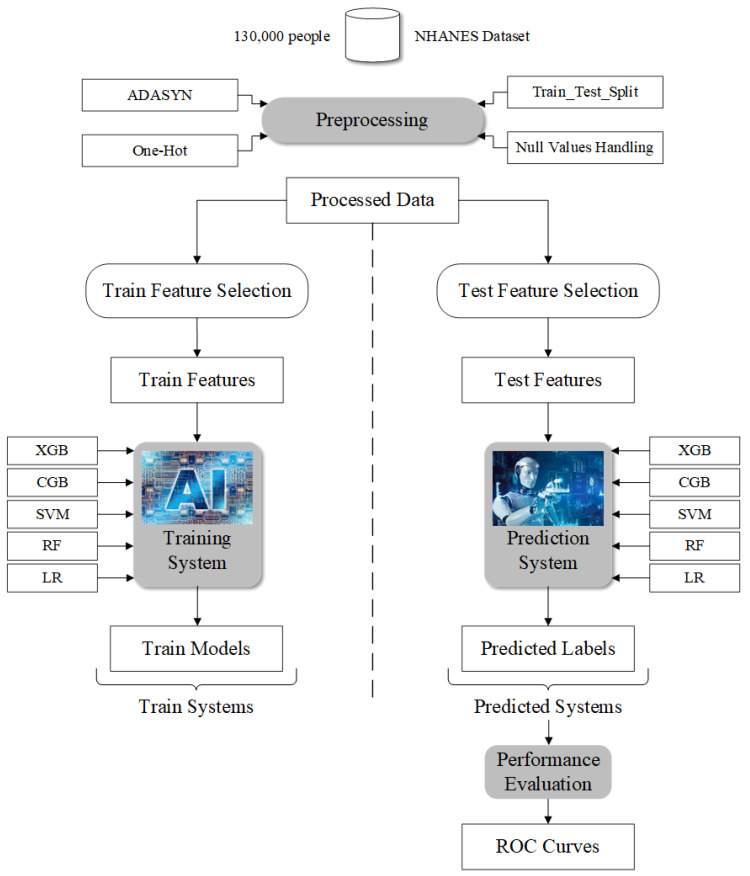
Data curation and machine learning process.

Figure 1 shows the design flow of this study. We first curated the dataset. Before training, we used One-Hot encoding to process categorical features and pad the null values. Also, due to the obvious class-imbalanced characteristics of our data, we used the SMOTE-NC algorithm to process the data to make it appear class-balanced. Finally, we used the Train_Test_Split library in Python to split it into an 8:2 (train set: test set) ratio. After data processing, we used the processed data to select features and finally screened 18 features that contributed to the model. Finally, we used training and trained five different machine learning models (XGB, CGB, SVM, RF, LR). After model training, we use the trained model to make predictions on the test set and evaluate the model through various metrics.

Figure 1 shows the process of data curation and machine learning.

## 3. Results

In our study, we divided the dataset into two types of samples with different labels: positive data samples with diabetes and negative data samples with diabetes. We applied the feature extraction method of minimizing the amount of information with the aim of selecting the model with the smallest AIC. AIC can improve a model’s reasonable degree (maximum likelihood) and incorporate a penalty term to reduce the model parameters as well as the possibility of overfitting. The filtered features are then used to train five classifiers in order to generate the corresponding five machine-learning models. These five models were then used on the test set to predict the lifestyle-diabetes link. Section 3.1. demonstrates how to filter features using the minimization of the informativeness criterion, while Section 3.2. demonstrates how to balance the data using the SMOTE-NC method. Finally, in Section 3.3., the performance of the machine-learning model is reported. Table 3 and Table 4 present a descriptive analysis of the data. Table 3 is the descriptive analysis of continuous data, and Table 4 is the descriptive analysis of categorical data.

### 3.1. Feature Selection

The two driving principles for selecting the best model should be the maximization of the likelihood function and the reduction of the number of unknown model parameters. The greater the likelihood function value, the better the model fits. However, we cannot merely judge the model’s quality by how well it fits since this introduces additional unknown parameters into the model, and as the model becomes more sophisticated, overfitting will occur. A good model should thus be a thorough, optimal arrangement of fitting accuracy and the number of unidentified parameters. Therefore, in the feature selection process, we employ the Akaike Information Criterion. Before comparison, the code creates several potentially different variable models and then compares them using AIC. The lower the AIC score, the better the model fit.

In the AIC approach, this paper adopts the forward regression method. The forward regression method is based on inserting independent variables used in the research into the model one by one, and the AIC value is used will be used to assess the model each time a new variable is included. When the newly added variable can no longer improve the model, it will be removed, and so on until all variables are traversed.

The realization idea of the Python code is

(1) Select an AIC variable to calculate and use it as the standard for comparing AIC values;
(2) Select a new variable from all features to be included in the model and calculate the AIC value. A program loop is required n times to obtain n different AIC values if there are n variables;
(3) The AIC value of each new variable is calculated and compared to the previous AIC value. If the AIC value decreases, the variable is included; otherwise, the variable is excluded;
(4) Repeat steps 2 and 3 until the addition of variables does not improve the model.

Table 2 shows the feature selecting order ranked by AIC score. It can be observed that as the number of characteristics rises, as well as the performance. The AIC value after incorporating 18 variables into the model is 15,460.8. Simultaneously, introducing new variables will not be able to reduce the model’s AIC value. Therefore, these 18 models (including variables) yield the highest-performing machine-learning models.

### 3.2. Balanced vs. Unbalanced Performance Parameters

Table 5 and Table 6 compare training machine-learning models for diabetes prediction using unbalanced and balanced data. It can be seen that the result parameters (such as accuracy and sensitivity) of all machine-learning models have met the standard, and the difference is relatively small but very low in specificity and very different; this is because the data in the dataset was unbalanced before data balancing using SMOTE-NC, the majority of the sample data are from data without diabetes (the ratio of people with diabetes to those without diabetes is 2587:11,679), and the specificities of the five machine-learning model classifiers were 13.8%, 36.8%, 18.9%, 11.8%, and 21.2%, respectively. After balancing the data with SMOTE-NC (the ratio of people with diabetes to those without diabetes is 11,739:11,679), we trained and re-evaluated the machine learning-based diabetes prediction, this time with the same training data in two different classes; after training and prediction, we observed that the machine-learning model’s accuracy and sensitivity decreased significantly, while its specificity increased significantly. The specificity for CGB is nearly constant, which may be due to the randomization in the implementation of SMOTE-NC. Although the machine-learning model’s overall prediction performance suffers slightly, its specificity improves significantly, reducing the model—misdiagnosis rate significantly.

### 3.3. Feature Importance of Machine-Learning Model

**Figure 2:**
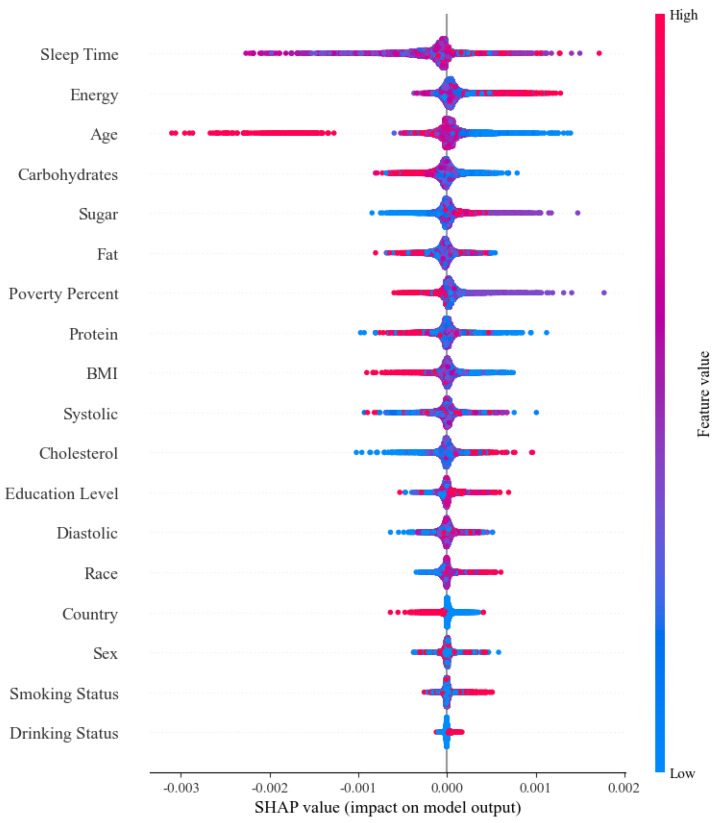
Feature importance of a model based on SHAP values.

Figure 2 depicts the relative importance of features utilized in the Catboost model to forecast features. We use Catboost to explain the relative importance of features for two reasons: (1) Catboost offers reasonably high performance in most performance metrics; (2) Catboost is a number-based classification model, therefore it can be explained using SHAP’s (Shapley Additive exPlanation) TreeExplainer (a fast and accurate algorithm).

The SHAP model is a versatile method that provides model interpretability. It can be interpreted both globally and locally. SHAP is an ex-post explanation method. Its core idea is to calculate the marginal contribution of features to the model output and then explain the “black box model” from the global and local levels. For each predicted sample, the SHAP model produces a predicted value called the SHAP value. It uses a value to measure the importance of a feature to the model.

In the figure’s left column, the variables used in the model training and prediction are displayed from top to bottom based on the size of the SHAP value (feature importance). The SHAP value represents the distribution of different parameters’ influence on the model construction. Red represents higher parameter values in the illustration, whereas blue represents lower parameter values.

The three most important predictors of diabetes are as follows: Sleep Time, Energy, and Age. Further exploration of the SHAP values of different tree ensemble models (XGBoost, CatBoost, RandomForest) shows that the four most important features remain unchanged across machine-learning models.

Figure 2 shows the ranking of the importance of each feature in the prediction of diabetes by the machine learning model. The 18 features are ranked in descending order of importance: Sleep Time > Energy > Age > Carbohydrates > Sugar > Fat > Poverty Percent > Protein > BMI > Systolic > Cholesterol > Education Level > Diastolic > Race > Country > Sex > Smoking Status > Drinking Status. It shows that in our machine-learning model, Sleep Time, Energy, and Age are the feature variables that contribute the most to the machine-learning model, while Sex, Smoking Status, and Drinking Status are the feature variables that contribute the least to the machine-learning model.

### 3.4. The Performance of the Machine-Learning Model

Table 7 demonstrates that the accuracy of the five machine-learning models is more significant than 67%, indicating that the models can make sound predictions on the data in the test set. However, since the test set has a class imbalance, sensitivity (miss rate) and specificity (misdiagnosis rate) should also be considered when considering accuracy. The precision-weighted scores (recall rates) of the five machine-learning models in this study were 82%, 80%, 81%, 81%, and 79%, respectively, while the sensitivity-weighted scores were 82%, 71%, 67%, 78%, and 69%. The values of sensitivity-weighted scores indicated that the five machine-learning models could predict negative samples well while maintaining a low missed diagnosis rate. Simultaneously, the specificity of the five machine-learning models was lower than other performance indicators, which were 51.9%, 35%, 33.3%, 43.9%, and 33.1%, respectively. The reason for this may be the small number of negative samples in the test set.

**Figure 3:**
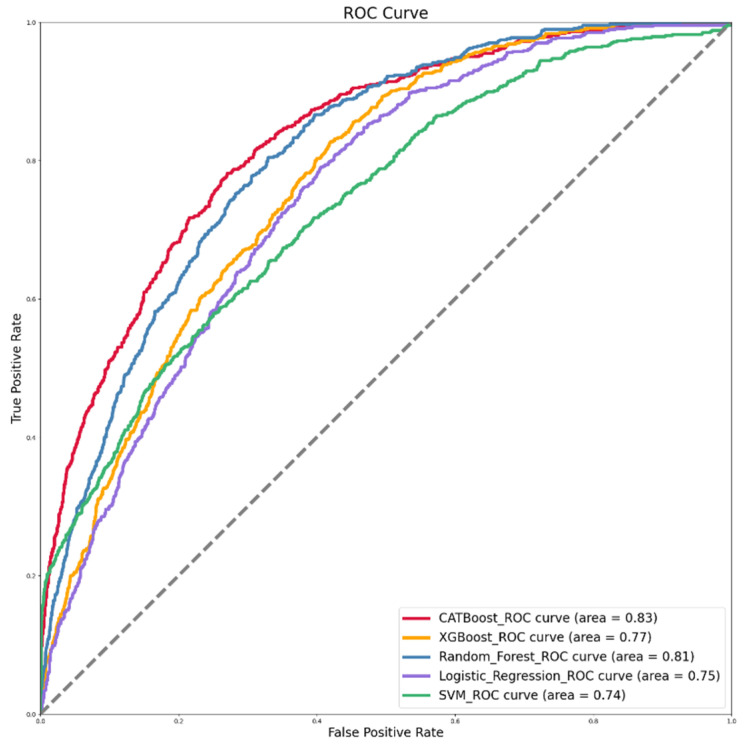
ROC curve figure of five machine-learning models.

Figure 3 depicts the ROC curves of the five machine-learning models, with AUC values ranging between 0.5 and 1. The larger the value of AUC, the better the model’s performance. Since AUC (ROC) is resistant to the uneven distribution of positive and negative samples in the test set, when the distribution of positive and negative samples in the test set changes, the ROC curve can remain stable, allowing the ROC curve to be well applied in this study’s dataset. As shown in Figure 3, the ROC curve areas of the five models are ranked as CATBoost > RF > LR > XGBoost > SVM.

Among the five machine-learning models, the best-performing model is CATBoost, followed by RF, LR, XGBoost, and SVM. CATBoost outperforms the others, whereas SVM performs the worst. On each performance criterion, the five machine-learning models achieved satisfactory and reliable results.

## 4. Discussion

### 4.1. Main Findings

Using five machine-learning models (CATBoost, XGBoost, RF, LR, and SVM), we attempted to predict diabetes based on lifestyle-related variables. We preprocessed the input data before training the model and used the mean to impute data with <5% missing values. In addition, we used SMOTE-NC to class-balance the data (the number of negative samples was much larger than the number of positive samples) to prevent the machine-learning model from outputting a single negative conclusion when making predictions when our dataset is class-imbalanced. Ultimately, we found that the performance ranking of the five machine-learning models was CATBoost > RF > LR > XGBoost > SVM. CATBoost has the best performance indicators among the five machine-learning models, with an AUC of 0.83 and an accuracy rate of 82.1%. Therefore, CATBoost may be employed as the best model for diabetes prediction for individuals in the prediction of machine-learning models based on or even modality variables.

### 4.2. Model Performance 

Previous studies have attempted to build machine-learning models for predicting diabetes. However, most previous studies employed random forests, logistic regression, k-nearest neighbors, and other machine learning models based on bagging algorithms to predict diabetes [45,46,47,48]. For example, Hu et al. [47] built a diabetes prediction model for adolescents using logistic regression and Gradient Boosted Tree and finally obtained a machine-learning model with an RUC of 71%. Krishnamoorthi [49] et al. An intelligent framework for diabetes prediction was constructed by applying four machine learning methods, with an RUC of 0.8 and final prediction accuracy of 83%. Although previous researchers have achieved good model results, few machine learning models for diabetes prediction based on the Boosting algorithm are still few. For example, Kumar et al. used CatBoost, logistic regression, support vector machines, and artificial neural networks to predict the probability of diabetes in gestational women and finally got an AUC of 0.86. Zhang et al. used logistic regression, support vector machines, random forests, and Catboost and Xgboost to predict childhood insulin resistance and obtained an AUC of 0.85. Using the Boosting algorithm to develop a model can better solve the problem of gradient bias and prediction offset, thereby reducing the occurrence of overfitting and improving the accuracy and generalization ability of the algorithm [45,46,47,48]. Compared with previous studies, our study optimized the previous researchers’ diabetes prediction model, which achieved higher AUC and accuracy rates on easy access to characteristic variables.

### 4.3. Model Features

As characteristic factors in our study, we selected demographics data and 18 lifestyle data from NHANES. Gender, age, race, country of birth, education level, poverty ratio, BMI, energy, protein, carbohydrates, sugar, total fat, cholesterol, smoking status, sleep duration, alcohol consumption, and systolic and diastolic blood pressure were among them. Most previous researchers [50,51,52] included biochemical indicators as an essential variable in their diabetes-prediction models. Lifestyle-related indicators have better collectability and de-aggression than biomarkers. Therefore, the model we established is independent of biochemical indicators, and our goal is to construct a practical and user-friendly screening model.

Figure 2 depicts the relative importance of various variables, which is consistent with past reports [53], with sleep duration, daily nutrient intake (such as energy, carbohydrates, fat, etc.), and age being the most important variables influencing model design. Furthermore, according to Civeira’s research [54], age ranks third in feature importance as an essential demographic variable.

### 4.4. Model Advantage

In the diabetes domain, diabetes datasets are often class-imbalanced datasets (a large number of people without diabetes and a small number of people with diabetes). Therefore, in contrast to earlier studies [46], we employed the SMOTE-NC method to deal with imbalanced class data. In contrast to prior research that did not use class imbalanced data, our machine-learning model could reduce missing and misdiagnosis rates. In addition, having too many variables in the model makes it heavy and slow. The significant increase in the data dimension will in-crease the complexity of the classification model, as well as the phenomenon of overfitting [32]. Feature selection plays an important role in traditional [55,56] and deep machine learning [57]. Thus we utilized the quantifiable AIC forward [33] propagation metric to filter the variables and eliminate the variables that contributed the least to the model.

### 4.5. Prospective to the Future

In the future, our research will be more inclined to the field of deep learning, combining big data samples with deep learning models. In addition, we will further increase the sample size and strive to optimize the model further to have better model prediction performance.

## 5. Conclusions

Although researchers have previously developed diabetes-prediction models, the majority of these models rely on standard statistical approaches. With the continuous development and refinement of machine learning in recent years, it has been increasingly applied in the healthcare community. This study demonstrates the potential of machine-learning methods for predicting diabetes using lifestyle-related data. We used NHANES data from 17,833 respondents and employed AIC forward propagation to filter out 18 lifestyle variables for model training. After model training, we evaluated the model using AUC. Finally, the performance ranking of the five machine-learning models we assessed was CATBoost > RF > LR > XGBoost > LR. Among these, the CATBoost model achieved an accuracy of 82.1% and an AUC of 0.83. Our cumulative results indicate that the proposed model can better predict diabetes using lifestyle-related indicators.

## Figures and Tables

**Table 1:** Detailed variable description.

Type of Data	Variable Symbol	Variable Description
Demographics Data	RIAGENDR	Gender of the participant
	RIDAGEYR	Age in years at screening
	RIDRETH	Race/Hispanic origin
	DMDBORN	Country of birth
	DMDYRUSZ	Length of time in US
	DMDEDUC	Education level
	DMDMATZ	Marital status
	RIDEXPRG	Pregnancy status at exam
	INDFMPIR	Ratio of family income to poverty
Dietary Data	DR1TKCAL	Energy (kcal)
	DR1TPROT	Protein (gm)
	DR1TCARB	Carbohydrate (gm)
	DR1TSUGR	Total sugars (gm)
	DR1TFIBE	Dietary fiber (gm)
	DR1TTFAT	Total fat (gm)
	DR1TCHOL	Cholesterol (mg)
Examination Data	BMI	Body Mass Index
	BPXOSY	Systolic
	BPXODI	Diastolic
Laboratory Data (outcome)	LBDGLUSI	Fasting Glucose (mmol/L)
Questionnaire Data	ALQ111	Ever had a drink of any kind of alcohol
	PA	Physical Activity
	SMQ020	Smoked at least 100 cigarettes in life
	SLD012	Sleep hours-weekdays or workdays
	MHDS	Mental Health-Depression Screener

**Table 2:** Process table for feature selection with reference to the Akaike Information Criterion.

Variable	AIC
RIDAGEYR	16,753.5
BMI	16,096.1
DMDEDUC	15,866.9
BPXOSY	15,704.5
RIAGENDR	15,636.9
ALQ111	15,599.7
DR1TSUGR	15,580.7
DR1TTFAT	15,545.9
INDFMPIR	15,521.6
SLD012	15,511.7
SMQ020	15,502.4
DMDBORN	15,494.7
BPXODI	15,490.8
DR1TKCAL	15,480.3
DR1TPROT	15,473.3
DR1TCARB	15,463.5
DR1TCHOL	15,461.2
RIDRETH	15,460.8

**Table 3:** Descriptive analysis of continuous data (mean ± standard deviation).

Variable Name	M ± SD
RIDAGEYR	50.0 ± 18.0
INDFMPIR	2.5 ± 1.6
BMI	29.4 ± 7.0
DR1TKCAL	2092.6 ± 967.6
DR1TPROT	80.6 ± 41.7
DR1TCARB	249.6 ± 122.7
DR1TSUGR	109.5 ± 75.3
DR1TTFAT	81.2 ± 45.9
DR1TCHOL	299.1 ± 240.1
SLD012	7.2 ± 2.4
BPXOSY	124.1 ± 19
BPXODI	70.2 ± 12.4

**Table 4:** Descriptive Analysis of Categorical Data.

Variable Name	Label	Frequency
RIAGENDR	Male	8858
	Female	8975
RIDRETH	Mexican American	2783
	Other Hispanic	1635
	Non-Hispanic White	7777
	Non-Hispanic Black	3699
	Other Race-Including Multi-Racial	1939
DMDBORN	Born in 50 US states or Washington	13,261
	Others	4572
DMDEDUC	Less than 9th grade	1978
	9–11th grade (Includes 12th grade with no diploma)	2407
	High school graduate/GED or equivalent	4028
	Some college or AA degree	5291
	College graduate or above	4129
SMQ020	yes	8095
	no	9738
ALQ111	yes	13,696
	no	4137

**Table 5:** Performance (accuracy and precision) of the 5 classifiers on diabetes-prediction balanced and unbalanced data.

Classifier	Accuracy (%)			Precision (%)		
	Without SMOTE-NC	With SMOTE-NC	% Change	Without SMOTE-NC	With SMOTE-NC	% Change
XGB	83%	70.8%	−12.2%	85%	80%	−5%
CGB	85.1%	82.1%	−3%	84%	82%	−2%
RF	84.4%	78.4%	−10%	85%	81%	−4%
LR	81.5%	68.9%	−12.6%	77%	79%	+2%
SVM	83.9%	67%	−16.9%	83%	81%	−2%

**Table 6:** Performance (specificity and sensitivity) of the 5 classifiers on diabetes-prediction balanced and unbalanced data.

Classifier	Specificity (%)			Sensitivity (%)		
	Without SMOTE-NC	With SMOTE-NC	% Change	Without SMOTE-NC	With SMOTE-NC	% Change
XGB	13.8%	35%	+21.2%	84%	71%	−13%
CGB	36.8%	51.9%	+15.1%	85%	82%	−3%
RF	18.9%	43.9%	+25%	84%	78%	−6%
LR	11.8%	33.1%	+21.3%	81%	69%	−12%
SVM	21.2%	33.3%	+12.1%	84%	67%	−17%

**Table 7:** Classifier performance of five classifiers in predicting diabetes.

Classifier Performance Parameters for Diabetes Prediction						
	Accuracy (%)	Precision (%)	Sensitivity (%)	Specificity (%)	F1 Score (%)	AUC (0–1)
CGB	82.1%	82%	82%	51.9%	82%	0.83
XGB	70.8%	80%	71%	35%	74%	0.77
SVM	67%	81%	67%	33.3%	71%	0.74
RF	78.4%	81%	78%	43.9%	79%	0.81
LR	68.9%	79%	69%	33.1%	72%	0.75

## Data Availability

All data in this article can be downloaded at https://www.cdc.gov/nchs/nhanes/index.htm, accessed on 1 September 2022.
